# Short linear motif acquisition, exon formation and alternative splicing determine a pathway to diversity for NCoR-family co-repressors

**DOI:** 10.1098/rsob.150063

**Published:** 2015-08-19

**Authors:** Stephen Short, Tessa Peterkin, Matthew Guille, Roger Patient, Colin Sharpe

**Affiliations:** 1 Institute of Marine Sciences, School of Biological Science, University of Portsmouth, Portsmouth PO1 2DY, UK; 2 The Weatherall Institute of Molecular Medicine, University of Oxford, John Radcliffe Hospital, Headington, Oxford OX3 9DS, UK; 3 Institute of Biomolecular and Biomedical Science, School of Biological Sciences, University of Portsmouth, Portsmouth PO1 2DY, UK; 4 European Xenopus Resource Centre, University of Portsmouth, St Michael's Building, Portsmouth PO1 2DT, UK

**Keywords:** co-repressor, NCoR family, alternative splicing, short linear motifs, pathway to diversity, isoforms

## Abstract

Vertebrate NCoR-family co-repressors play central roles in the timing of embryo and stem cell differentiation by repressing the activity of a range of transcription factors. They interact with nuclear receptors using short linear motifs (SLiMs) termed co-repressor for nuclear receptor (CoRNR) boxes. Here, we identify the pathway leading to increasing co-repressor diversity across the deuterostomes. The final complement of CoRNR boxes arose in an ancestral cephalochordate, and was encoded in one large exon; the urochordates and vertebrates then split this region between 10 and 12 exons. In *Xenopus*, alternative splicing is prevalent in NCoR2, but absent in NCoR1. We show for one NCoR1 exon that alternative splicing can be recovered by a single point mutation, suggesting NCoR1 lost the capacity for alternative splicing. Analyses in *Xenopus* and zebrafish identify that cellular context, rather than gene sequence, predominantly determines species differences in alternative splicing. We identify a pathway to diversity for the NCoR family beginning with the addition of a SLiM, followed by gene duplication, the generation of alternatively spliced isoforms and their differential deployment.

## 1. Introduction

Vertebrates may be intuitively described as more complex than invertebrates, but the molecular basis for this distinction, and the pathways by which it is achieved, are less apparent. Because total gene counts are often comparable, it has been suggested that increases in the number and type of regulatory DNA elements, combined with an increased diversity in the composition of the transcription factor complexes with which they interact, may begin to account for the increasingly complex patterns of gene expression seen over evolutionary time (reviewed in [1,2]). In contrast, even small changes to the sequence and structure of transcription factors themselves are likely to disrupt their activity and have deleterious effects. The recent identification, however, of short linear motifs (SLiMs), defined as functional peptide modules 3–10 amino acids in length [3,4] which act as modular components within a larger protein, points to these as independent targets for evolutionary change, because the gain, loss or alteration of one motif is less likely to compromise the activity of others [5]. In addition, many genes use multiple promoters and alternative splicing to make several transcripts from one gene that can then be translated into isoforms with distinct functions [6,7]. Using alternative splicing to generate isoforms that differ in their complement of SLiMs will generate related proteins with diverse functions that may contribute to organismal complexity [3].

Vertebrate nuclear co-repressors NCoR1 and NCoR2, also known as silencing mediator for retinoid or thyroid-hormone receptors (SMRT), are large proteins whose genes are derived from a common ancestor. Co-repressor activity is reflected in their structure, in which the 50 amino acid amino-terminal SANT domains (named after Swi3, Ada2, NCoR and TFIIIB) [8] are core to regions that interact with histone deacetylases to put chromatin into a compact, transcriptionally inactive state [9–13]. Sequences that mediate the interaction with the nuclear receptor transcription factors, however, are found as SLiMs, termed co-repressor for nuclear receptor (CoRNR) boxes, embedded within a carboxy-terminal region that lacks significant structural organization [14–18]. Type II nuclear receptors, such as the retinoid receptors, bind DNA as heterodimers with a common, RXR partner [19–22], and each co-repressor is thought to interact with a nuclear receptor dimer [18,22,23]. To achieve this, NCoR1 uses any two of its three CoRNR boxes to bind directly to the receptors, but only in the absence of the receptor's ligand, such as retinoic acid [14–17,24,25]. The human, mouse and *Xenopus* NCoR2 genes also encode three CoRNR boxes, equivalent to those in NCoR1 but, through alternative splicing, produce protein isoforms with variable numbers of these motifs [24–29]. The co-repressors bind to a wide range of nuclear receptors and the different *in vitro* affinities of the CoRNR boxes for nuclear receptors and their distribution between the NCoR2 isoforms demonstrate that alternative splicing generates diverse isoforms that preferentially interact with specific subsets of nuclear receptors [25,28–31].

Each co-repressor acts as a platform for the assembly of multi-protein complexes [32,33] that actively repress a remarkably wide range of transcription factors including most, if not all, of the type II nuclear receptors and, among others, the transcription factors Pit1, PLZF, Bcl-6, NFκB, SRF, CBF-1 and ETO (reviewed in [28]). Not surprisingly, NCoR1 and NCoR2 have been implicated in diverse biological processes. NCoR1 knockouts in mice have lethal defects in erythropoiesis [34], while NCoR2 knockouts die from defects in cardiac development [35,36]. NCoR1 and NCoR2 also affect embryonic development [24,37], neural stem cell differentiation [35,38], homeostasis [39], oxidative metabolism and ageing [40], adipocyte differentiation [31] and embryonic blood formation [41]. Altered interactions between the co-repressors and mutated retinoid receptors underlie acute promyelocytic leukaemia [42–44] and primary myelofibrosis [45], while NCoR2 has been implicated in the progression of glioblastoma in animal models [46].

In most vertebrates, the 3′ part of the gene encoding the carboxy-terminal region of each co-repressor is divided between 10 exons. In NCoR2, this structure underpins alternative splicing to generate isoforms with different numbers of CoRNR boxes. For example, exon 37 encodes a CoRNR box, but the use of an internal splice donor generates an isoform lacking this motif. The capacity for exon 37 alternative splicing in NCoR2 is conserved between *Xenopus*, mice and humans [27]. While both exon 37 isoforms are found at roughly equivalent levels in *Xenopus* tissues, in mice the outcome of exon 37 alternative splicing is tissue specific, with the CoRNR box-containing isoform (37b+) predominant in the brain and the CoRNR box excluded isoform (37b−) found in most tissues [27,29]. Unlike NCoR2, there is no detectable alternative splicing of this exon in *Xenopus* NCoR1, but a distinct isoform has been reported in mammals [27,28].

Significant differences in function between NCoR2 isoforms have been demonstrated *in vitro* [25,29,30]. The exclusion of NCoR2 exon 37b *in vivo*, during *Xenopus* development, results in embryos with deformed heads, disturbed axon guidance and the repression of some early thyroid hormone responsive genes, indicating this alternative splicing event is significant for embryogenesis [24]. In addition, mice engineered to express NCoR2 with defective CoRNR boxes show a range of mutant phenotypes [40,45,47]. These results indicate that the CoRNR boxes are not redundant, because a full complement is required for normal function.

The gain and loss of SLiMs in proteins involved in transcriptional control is a significant mechanism in vertebrate evolution [5]. In addition, the direct correlation between intrinsically disordered regions (IDRs) and alternatively spliced exons [48], combined with the frequent presence of SLiMs in IDRs, indicates a mechanism by which the assortment of SLiMs between tissue-specific isoforms can contribute to functional complexity at the level of the cell (reviewed in [3]). Using the nuclear co-repressors as a test case, we extend this concept from cells to organisms by demonstrating a transformative increase in the diversity of these proteins from sea urchin to frog. The pathway to diversity, involving progressive SLiM acquisition, augmented by a striking exon fragmentation and the deployment of alternatively spliced isoforms, defines a direct mechanism by which the complexity of interactions of a family of transcription-associated proteins is enhanced over evolutionary time.

## 2. Results

### 2.1. The acquisition of short linear motifs

**Figure 1.**
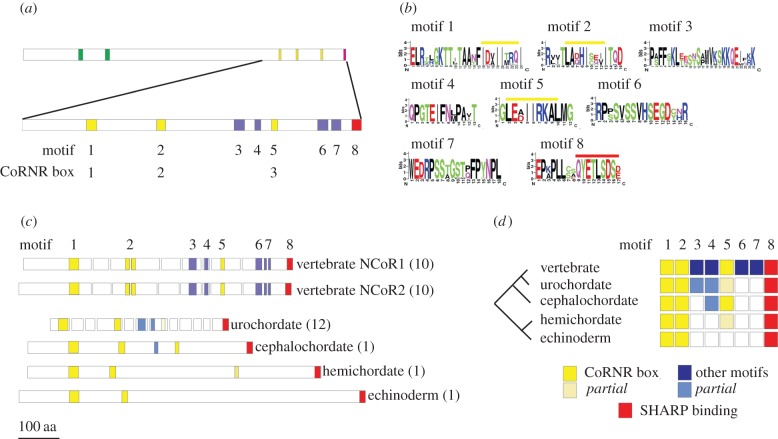
The NCoR-family conserved motifs and exon structure. (*a*) The NCoR-family proteins in the vertebrates typically contain two SANT domains (green bar), followed by three CoRNR boxes, nuclear receptor interaction motifs (yellow bars) and a carboxy-terminal SHARP-binding motif (red bar). Alignment of vertebrate NCoR1 and NCoR2 sequences identifies a further four conserved motifs (blue bars, lower diagram). Full sequence alignments are in the electronic supplementary material, figure S1. (*b*) Identity of conserved vertebrate sequences using the motif notation. Yellow bars overlie the consensus CoRNR box motif L/I.x.x.I/H.I.x.x.x.I/L [50,51] that is embedded in each of motifs 1, 2 and 5. The C-terminal SHARP-binding sequence is overlined in red as part of motif 8. (*c*) Exon organization of the 3′ end of representative NCoR-family genes. The regions encoding the motifs have been mapped onto the relevant exons maintaining the colour scheme in (*a*). In parentheses is the number of exons in this region of the gene. The C-terminal motifs are encoded by one large exon encoding 843 amino acids in the sea urchin, but 12 exons encoding 365 amino acids in the sea squirt. (*d*) Summary of C-terminal motif acquisition across the representative deuterostome panel. All contain motifs 1,2 (CoRNR boxes 1 and 2) and 8, but motif 5 (the third CoRNR box) is not present in the echinoderm and incomplete in the hemichordate and urochordate. Two of the four vertebrate specific motifs (3 and 4) are represented by partial motifs in the urochordate and cephalochordate (motif 4 only). Sequence alignments of the motifs are in the electronic supplementary martial, figure S2.

The vertebrate paralogues NCoR1 and NCoR2 are defined by two SANT domains [9–13], three CoRNR box motifs that mediate interactions with nuclear receptors [14–17,24,26] and a carboxy-terminal domain that interacts with SHARP, a transcriptional repressor [49] (figure 1*a*). Alignment of vertebrate NCoR1 and NCoR2 C-terminal sequences identified four additional conserved short motifs (figure 1*a,b* and electronic supplementary material, figure S1) that will be targets for future functional analysis.

SLiMs, such as CoRNR boxes, in the carboxy-terminal region mediate many of the interactions of the NCoR-family co-repressors with transcription factors [25,28–31]. Because additional isoform diversity, particularly in NCoR2, is generated by alternative splicing of the primary transcript in this region, we next looked at the organization of exons encoding the C-terminal interaction domains and mapped the SLiMs to their encoding exons (figure 1*c*). The organization of the paralogues is highly conserved in vertebrates with most having 10 exons, from that encoding the first CoRNR box (exon 37) to the stop codon (exon 46). An exception is zebrafish NCoR2, which lacks exon 38, although many other actinopterygians have the standard vertebrate organization (data not shown).

The presence of the domains and motifs was used to confirm the annotation of NCoR-family proteins encoded in invertebrate deuterostome genomes and identified that a representative echinoderm, *Strongylocentrotus purpuratus* (sea urchin), hemichordate, *Saccoglossus kowalevskii* (acorn worm), cephalochordate, *Branchiostoma floridae* (amphioxus), and urochordate, *Ciona intestinalis* (sea squirt) each encodes one NCoR-family co-repressor (figure 1*a* and see electronic supplementary material, table S1 for a list of identities). In *Ciona*, the C-terminal region is encoded by 12 exons, though only a few of the exons have boundaries in common with those in the vertebrates (figure 1*c*). More striking are *Strongylocentrotus purpuratus* (sea urchin), *Saccoglossus kowalevskii* (acorn worm) and *Branchiostoma floridae* (amphioxus) NCoR-family genes, which each encode the C-terminal region in just one exon (figure 1*c*).

Mapping the conserved C-terminal motifs from the vertebrates to this collection of invertebrate deuterostome NCoR-family proteins (figure 1*c*) overall suggests a progressive acquisition of motifs (figure 1*d*; electronic supplementary material, figure S2 for sequences). Interestingly, the sea urchin lacks the third, most C-terminal CoRNR box seen in vertebrates, while in the acorn worm it is incomplete, lacking the conserved C-terminal leucine or isoleucine, a distinctive feature of the CoRNR box. To determine if this third CoRNR box is functional would require biochemical binding assays, but it is worth noting that acorn worm CoRNR boxes 1 and 2 have complete motifs indicating that the full sequence can interact with acorn worm nuclear receptors. The change in CoRNR box complement is reminiscent of the acquisition of a similar SLiM in the *Ftz* gene across an insect phylogeny [52]. Although the common deuterostome ancestor may, alternatively, have had three CoRNR boxes, with a subsequent loss in the Ambulacraria, the ability of the vertebrate NCoR-family co-repressors to interact efficiently with the wide range of nuclear receptors will have been enhanced by the presence of a third CoRNR box in the common ancestor of the cephalochordates and the vertebrates, because the identity of the individual CoRNR boxes drives the interactions of the co-repressors (reviewed in [28]).

### 2.2. The loss of splicing potential in the 3′ region of the NCoR1 gene

We have previously shown that while *Xenopus* NCoR2 has 16 C-terminal isoforms, generated by the alternative splicing of four exons, despite having the same gene organization, *Xenopus* NCoR1 has a single isoform [26]. There are two possible explanations: first, that NCoR2 gained the capacity for alternative splicing or second, that NCoR1 lost the capacity for alternative splicing, subsequent to genome duplication, the latter being consistent with previous observations of alternative splicing and gene duplication [53]. To examine these possibilities, we have looked in more detail at exon 37, which in NCoR2 uses two splice donors to generate a long isoform (37b+) that contains motif 1 and a short isoform (37b−) that lacks this motif [26]. We have previously shown that an antisense morpholino oligonucleotide to the long isoform splice donor biases alternative splicing to produce predominantly the short 37b− isoform, without altering the overall level of NCoR2 transcripts either in the whole embryo or in the range of tissues examined. This experimental bias generates a distinct mutant phenotype, indicating the functional significance of exon 37 alternative splicing in embryonic development [24].

**Figure 2.**
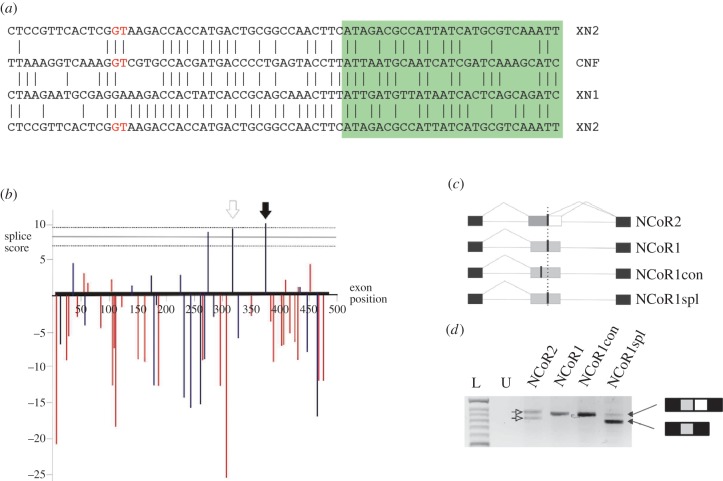
NCoR1 may have lost the capacity for alternative splicing in exon 37 after gene duplication. (*a*) Sequence alignments of *Xenopus* NCoR1 (XN1) and *Ciona* NCoR family (CNF) each with part of exon 37 of *Xenopus* NCoR2 (XN2). Vertical lines mark identical residues, the green box encodes CoRNR box 1. The sequences are conserved around the internal splice donor of NCoR2, except for the GT splice donor (red) that is a GA in NCoR1. (*b*) Plot of MaxEntScan splice score against position in exon 37 for all dinucleotides that can be changed by point mutation to a GT. Each bar represents the score for a nine-base sequence centred on the GT dinucleotide. The horizontal lines represent the mean and standard deviation calculated from all other characterized splice donors in the *Xenopus* NCoR1 gene. Blue bars mark splice donors that maintain the reading frame with exon 38 while red are out of frame. The black arrow marks the position of the site equivalent to the internal splice donor in NCoR2. The open arrow marks the position of the upstream potential splice donor used as a control for specificity. (*c*) Splicing constructs used to test the efficiency of the splice donors. Exon 37 from NCoR2 (grey and white box for exon 37b), NCoR1 and two point mutated forms of NCoR1 (grey boxes) were cloned along with flanking intron sequence between two human exons (black boxes) in the vector pTBNde1. A vertical dotted line marks the position of the internal splice donor equivalent to that found in NCoR2. Actual and potential GT splice donors are marked by a thick line. (*d*) RT-PCR analysis of transcripts from the splicing vector injected into *Xenopus* embryos. L marks the size ladder, U indicates uninjected control. The NCoR2 construct undergoes alternative splicing of exon 37 (open arrows), while the wild-type NCoR1 and the upstream specificity control (NCoR1con) do not, and produce a single band of the length expected for the inclusion of the entire exon 37. In contrast, the NCoR1 construct containing the point mutation at the equivalent internal site to NCoR2 (NCoR1spl) produces two bands (black arrows), the smaller representing the exclusion of exon 37b.

Alignment of the 3′ part of *Xenopus* exon 37 in NCoR1 and NCoR2 shows extensive sequence conservation, apart from the internal splice donor, which in NCoR1 is a GA rather than the active GT dinucleotide seen in NCoR2 (figure 2*a*). Including the equivalent region of the *Ciona* NCoR-family gene in the comparison (figure 2*a*) shows a GT at the corresponding position suggesting that the common ancestor of *Ciona* and the vertebrates had a potential splice donor dinucleotide.

Because an effective splice donor requires sequences in addition to the conserved GT [54], we next tested whether the presence of a GT, rather than the GA, at the internal site in NCoR1 can reconstitute an effective splice donor. First, we used the program MaxEntScan [55] to quantify the effectiveness, as splice donors, of the sequences surrounding all dinucleotides in NCoR1 exon 37 that could be converted to a GT by a single base change, and then compared these with the range of scores found for all other validated *Xenopus* NCoR1 exon splice donors. The average score for the confirmed splice donors is just over 8, and this approach identified three sites within exon 37 with a greater score, and these sites are predicted to form strong splice donors when the core dinucleotide is mutated to a GT. Of these three sites, one was at the GA corresponding to the internal splice donor in NCoR2 and the other two were approximately 50 and 90 bp further upstream (figure 2*b*).

To determine, experimentally, whether the sequence context of the equivalent site in NCoR1 reconstitutes an effective splice donor, we used site-directed mutagenesis to convert the GA to a GT and then cloned the exon, and flanking intron sequences, into the splicing minigene, pTBNde1 [56]. Because it is possible that any GT that has a surrounding sequence calculated to be a strong splice donor might permit alternative splicing, we addressed specificity using an NCoR1 exon 37 minigene in which the first predicted strong site upstream of the equivalent site was also converted to a GT (figure 2*c*).

Transcripts from embryos injected at the two-cell stage with a plasmid minigene were analysed at the neurula stage by RT-PCR (figure 2*d*). An NCoR2 exon 37 minigene recapitulated the pattern of splicing seen in the native gene producing two bands of similar intensity [26], as did the wild-type NCoR1 minigene, which gave one band corresponding to the inclusion of the full-length exon. In contrast, the minigene with the introduced splice donor, at the equivalent site to the internal splice donor in NCoR2, generated two bands indicative of alternative splicing, the stronger band associated with splicing from the introduced internal splice donor. An introduced GT at the calculated upstream site was inactive, because only the long form transcript, identical to that from the native NCoR1 minigene, was produced (figure 2*d*).

Although we cannot discount a sequence of events in which an effective splice donor context arose in the NCoR-family precursor, followed by the gain of the obligatory GT solely in NCoR2, the simpler explanation, given the presence of the equivalent GT in both *C. intestinalis* and *Ciona savignyi*, is that exon 37 alternative splicing arose in the precursor but was subsequently lost from NCoR1, by point mutation, following gene duplication.

### 2.3. The conservation of NCoR2 exon 37 alternative splicing

**Figure 3.**
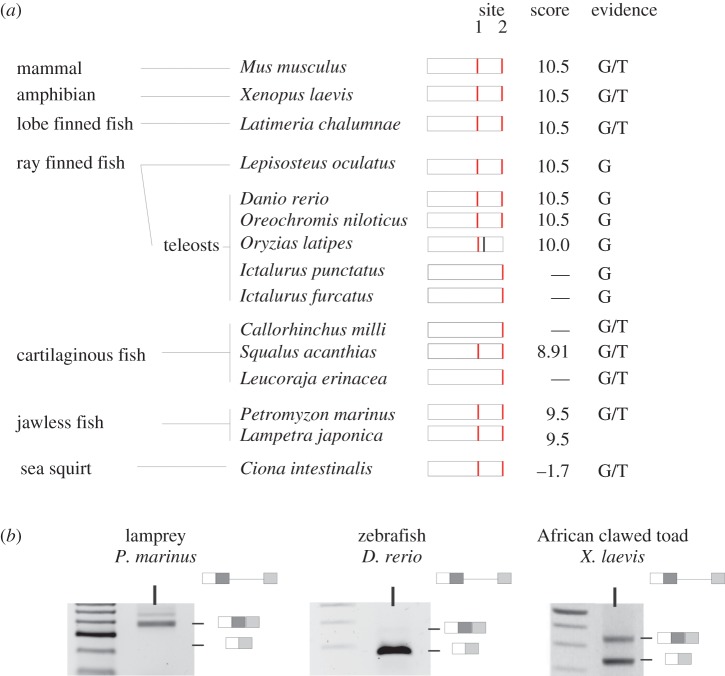
The conservation of NCoR2 exon 37 alternative splicing. NCoR2 exon 37 in *Xenopus* and mouse has two splice donors (red bars), site 1 is internal and site 2 is at the end of the extended exon. Exon 37b, between the two sites, encodes the CoRNR box 1 motif. (*a*) The potential site 1 GT splice donor is present in at least one representative of each vertebrate group and when present is part of a high-scoring consensus. A site 1 GT is present in the sea squirt, but is part of a poor consensus splice donor. (*b*) RT-PCR analysis of exon 37 alternative splicing across a range of species. The size of the PCR product generated from lamprey cDNA indicates that it includes exon 37b while that from 48 h zebrafish embryos corresponds predominantly to 37b–transcripts. *Xenopus*, in contrast, generates both isoforms as shown by the two bands in the RT-PCR.

Because the alternative splicing of NCoR2 exon 37 has been characterized in *Xenopus*, mouse and humans, and generates isoforms that differ in a functional CoRNR box motif [24], we next investigated the conservation of the internal splice donor across nine species of fish, two lampreys and *Ciona*. The position and splice donor strength of each GT dinucleotide across 115 bases of the 3′ part of NCoR2 exon 37, centred on the internal splice donor, was calculated by MaxEntScan (figure 3*a*).

While the NCoR-family gene in both *C. intestinalis *and* C. savigny* (sea squirts) has a GT at site 1, the equivalent position to the internal splice donor in *Xenopus* NCoR2, (figure 3*a*), it does not score well as a predicted splice donor and there is no published transcriptomic evidence for its use. The agnathostomes *Petromyzon marinus* (sea lamprey) and *Lethenteron japonicum* (Japanese lamprey) each have two paralogues and one, like NCoR1, lacks the equivalent internal splice donor while it is present in the second, where it is predicted to be a strong splice donor in the correct frame for productive splicing. EST data and limited RT-PCR analysis (figure 3*b*) for *Petromyzon marinus* (sea lamprey), though, suggest that the internal site is not commonly used.

Of three cartilaginous fish examined, only *Squalus acanthias* (dogfish) has site 1. Although the *Leucoraja erinacea* (little skate) has a site further upstream that is predicted to be an effective splice donor, the corresponding transcripts are not present in the reported transcriptome. Of the ray-finned fish, NCoR2 site 1 is present in four out of six genomes examined, being absent in two related catfish species. It is likely that the internal splice site donor is active in *Oryzias latipes* (medaka) because it is closely followed by an in-frame stop codon that would otherwise produce a truncated protein with compromised function (electronic supplementary material, figure S3). An assessment of 40-h post-fertilization (hpf) *Danio rerio* (zebrafish) embryos indicates that site 1 is predominantly used (figure 3*b*) and this is supported by EST data, but there is also a low level of the longer transcripts that use site 2. We next examined, in more detail, why the observed use of site 1 differs between species such as zebrafish and *Xenopus*, when the consensus splice-donor sequences are identical.

### 2.4. The acquisition of distinct patterns of alternative splicing in NCoR2 exon 37

**Figure 4.**
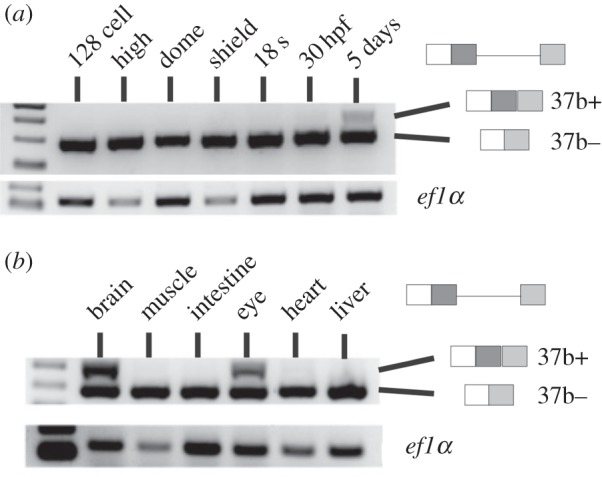
Evidence for the alternative splicing of exon 37 in zebrafish NCoR2. (*a*) RT-PCR analysis of RNA taken from a timecourse of zebrafish early development. The 37b− isoform is predominant and the 37b(+) isoform is only detectable in later development. (*b*) Adult zebrafish were dissected into different parts and extracted RNA assayed by RT-PCR. The 37b+ isoform is only detected in brain and eye suggesting the tissue-specific control of the alternative splicing of this exon. A similar pattern is seen in mice but not frogs [27].

During early development, zebrafish uses site 1 to produce solely the short (37b−) isoform, however a low level of the longer exon 37b+ transcripts can be detected by embryonic day 5 (figure 4*a*). This is likely to represent the production of NCoR2 exon 37b+ transcripts in neural tissue, as they are also found in the dissected brain and eyes of adult fish, but not in other tissues examined (figure 4*b*). This is similar to the tissue-specific pattern seen in mice [27]. Consequently, while both *Xenopus* and zebrafish use alternative splicing to generate NCoR2 exon 37 isoforms, strategies for isoform deployment differ in that the expression of both isoforms is widespread in *Xenopus*, but temporally, and spatially, regulated in zebrafish.

**Figure 5.**
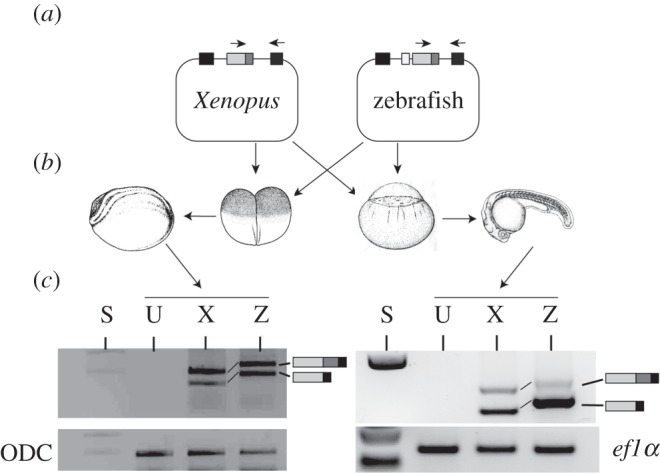
NCoR2 exon 37 splicing patterns are determined by cellular context. (*a*) *Xenopus* NCoR2 exon 37 (light grey box, dark grey box marks exon 37b) with approximately 250 base pairs of upstream and downstream flanking intron was cloned into the splicing vector pTBNde1 between two human exons (black boxes). Zebrafish exon 37 was similarly cloned but owing to the close proximity of exon 36 (white box) to exon 37 both exons, the intervening intron and 250 base pairs upstream of exon 36 and approximately 460 bp downstream were used. (*b*) Plasmids were injected into early stage *Xenopus* (left) and zebrafish (right) embryos and then grown to post-gastrula stages. (*c*) RNA extracted from the embryos was subject to RT-PCR using specific primers shown as small arrows in (*a*). *Xenopus* and zebrafish clones both gave two bands indicative of exon 37 alternative splicing in *Xenopus*. Injection into zebrafish embryos resulted in splicing primarily from the internal site to give the shorter 37b− isoform. This indicates that the pattern of alternative splicing is strongly influenced by the cellular context. S, size markers; U, uninjected; X, injected *Xenopus* construct; Z, injected zebrafish construct.

To determine whether the intrinsic sequence of the internal splice donor or its cellular context plays the greater role in determining the splicing pattern of NCoR2 exon 37, we generated splicing minigenes containing either zebrafish or *Xenopus* exon 37, together with flanking intron sequences, in the pTBNde1 minigene [56]. The minigenes were each injected into *Xenopus* embryos at the two-cell stage and splicing of the transcript from the minigene assayed by RT-PCR 1 day later (figure 5). Just as found in the endogenous gene, the *Xenopus* NCoR2 minigene produces two transcripts. The zebrafish NCoR2 minigene also now produces two transcripts in approximately equal amounts, in contrast to the total exclusion of the longer form seen for the endogenous gene in fish at an equivalent developmental stage. This suggests that the cellular context provided by the *Xenopus* embryos, rather than the intrinsic sequence of the splice donor, determines the outcome of exon 37 alternative splicing.

Because placing either minigene in a *Xenopus* context imitated the endogenous *Xenopus* pattern of alternative splicing, we next repeated the analysis, injecting the minigenes into zebrafish embryos. The *Xenopus* minigene again produced two bands, but this time with a significant bias towards the short form. This was even more pronounced for the zebrafish minigene (figure 5). Again, the pattern of alternative splicing of the minigenes mirrors that of the endogenous host gene indicating the importance of the cellular context.

*Xenopus* and zebrafish embryos differ in the way in which they regulate alternative splicing at exon 37. The sequences immediately adjacent to the internal splice donors are identical, as are those that surround the terminal splice donor, and internal and terminal sites have similar strength by MaxEnt Scan. One simple explanation is that the generation of both isoforms, seen in the *Xenopus* context, is determined predominantly by the balanced splice donor strengths, while zebrafish embryos either have a suppressor to inhibit the terminal splice donor or a splice-promoting protein to enhance the use of the internal donor that operates less efficiently on the sequences included in the *Xenopus* gene splicing construct. Later in development, the simple loss of expression of either type of factor in zebrafish neural tissue would result in the production of both isoforms in this tissue.

## 3. Discussion

The vertebrate nuclear receptor co-repressors, NCoR1 and NCoR2, play important roles in physiological [22,24,31,34–37,39–41] and pathological conditions [42–45] by interacting with a wide variety of transcription factors and other DNA binding proteins [28]. NCoR1 and NCoR2 interact with nuclear receptors via short sequence motifs called CoRNR boxes, located in the intrinsically disordered carboxy-terminal part of the co-repressor [14–18]. Alternative splicing, particularly in NCoR2, determines the complement of motifs in the protein and so generates diverse isoforms, each with specific binding capabilities [25,28–31]. As a result, NCoR1 and NCoR2 conform to a model where the selection of SLiMs by alternative splicing, from within an IDR of a protein, plays a significant role in the generation of functional diversity [3]. Here, we combine comparative and experimental approaches to analyse the origins of co-repressor diversity across the deuterostomes.

### 3.1. Diversity through motif acquisition

*Strongylocentrotus purpuratus* (sea urchin) has a single NCoR-family gene with only limited sequence homology to NCoR1 and NCoR2, but encoding two indicative SANT domains and three of the eight vertebrate NCoR-family motifs. These include two CoRNR boxes [14,50,51] that are typical SLiMs and a SHARP interacting motif at the carboxy-terminus of the protein. The remaining motifs may indicate regions that interact with other transcription factors or act as sites for post-translational modifications, such as phosphorylation, that, at other sites, are known to modulate the activity of the co-repressor protein *in vivo* [3,57–59]. In comparison, *Branchiostoma floridae* (amphioxus) produces a co-repressor with three complete CoRNR boxes. Increasing the number of motifs will increase the functional diversity of the co-repressor, because *in vitro* experiments using mouse or *Xenopus* proteins have shown that different CoRNR boxes have different affinities for specific nuclear receptors [14–17,25]. It is likely, however, that lifting repression by the ligand-dependent displacement of the co-repressor will be more significant than imposing repression by binding, because this mechanism would set ligand concentration thresholds for nuclear receptor activation that are dependent on the CoRNR box complement of the co-repressor. This concept is illustrated, in exaggerated fashion, in acute promyelocytic leukaemia, in which specific NCoR2 isoforms are displaced from the pathological RAR fusion protein at distinct concentrations of retinoic acid [44].

Changes to the *cis*-regulatory elements in the promoter of a transcription factor have been directly associated with evolutionary events [60]. Because most promoters are a collection of independent elements that each control a limited aspect of gene expression, a mutation in one element is likely to affect expression of the gene in only one component of its pattern. In contrast, mutations that affect the protein coding sequence of a transcription factor itself will tend to affect, often calamitously, the expression of all downstream targets [60]. The protein sequence changes seen in the NCoR family, however, illustrate how the consequences of changes to the protein coding sequence can be mitigated. By encoding functional SLiMs within IDRs, the gain or loss of a SLiM has an incremental effect, because the remaining functions of the protein are essentially maintained [5]. The insect Ftz protein, and its ability to interact with Ftz-F1, typically illustrates this interaction and involves a SLiM closely related to the core CoRNR box sequence [52].

### 3.2. Fragmentation of the invertebrate NCoR-family terminal exon

The entire C-terminal region, encoded by exons 37–46 in *Xenopus*, is encoded by a single exon in sea urchins and is predicted to have the same organization in acorn worms and amphioxus. In *C. intestinalis* (sea squirt), however, this part of the gene is divided into 12 exons and is consistent with chordate phylogeny, which predicts the tunicates, rather than amphioxus, are most closely related to the vertebrates [61]. A similar degree of discrepancy in exon number and exon boundary location between *C. intestinalis* and humans is seen in the huntingtin gene [62]. The trigger and mechanism for this remarkable and extensive fragmentation of the NCoR-family gene terminal exon is unknown.

### 3.3. Diversity through gene duplication

Across the deuterostomes analysed, a complement of two NCoR-family genes is first seen in the genome of the lampreys. Gene duplication opens the possibility for a form of subfunctionalization and neofunctionalization in which altered *cis*-regulatory events, alternative splicing and protein sequence changes happen within one paralogue on the background of an initially redundant second sequence [63,64]. Following gene duplication, the amino acid sequences of the paralogues have (apart from the identified motifs) diverged extensively in the C-terminal region such that NCoR1 and NCoR2 have less than 40% identity in humans (data not shown). Importantly, gene knockout studies in mice show that the two paralogues are no longer equivalent [22,34–36].

### 3.4. Diversity through alternative splicing: the case of NCoR2 exon 37

Comparisons between *Xenopus* NCoR1 and NCoR2 show a high degree of nucleotide sequence conservation across the latter half of exon 37. One difference, however, is the GT that forms the conserved core dinucleotide of the NCoR2 internal splice donor that is a GA in NCoR1. A GT at the equivalent position in the single gene in both *C. intestinalis* and *C. savignyi* suggests that the GT may be the ancestral form that changed to GA in the NCoR1 gene after duplication. A point change that restores the GT to the internal NCoR1 splice donor recovers the splicing activity of this site. There is more to the activity of this site, however, than just the dinucleotide and the immediate surrounding sequence, because the introduction of a GT upstream in the same exon, that generates a site predicted to be an efficient splice donor, is inactive in *Xenopus* embryos. Su *et al.* [53] have suggested that the loss of pre-existing alternative splicing in one paralogue, and the generation of more diversity in the other, may not be uncommon, and this seems a plausible scenario for NCoR1 and NCoR2.

Alternative splicing at exon 37b varies the number of CoRNR boxes in NCoR2 and this has functional significance in *Xenopus laevis* embryonic development [24]. Unlike *Xenopus*, the equivalent exon in NCoR1 is alternatively spliced in mammals to generate isoforms with different numbers of CoRNR boxes, though from a different splice donor [28] (and see NM_001190440). This is consistent with the idea that alternative splicing of exons that contain SLiMs within an IDR is an efficient mechanism for the generation of isoforms with different activities that can progressively contribute to the complexity of the cellular functions during evolution [3,65].

### 3.5. Diversity through the deployment of alternative splicing

With two splice donors in exon 37, zebrafish has the capacity for alternative splicing, but in the early embryo uses only the internal splice donor, and so the resulting isoform excludes one of the CoRNR boxes. It is only later in development, and in the adult, that alternative splicing is deployed, but restricted to neural tissues (figure 4). In contrast, *Xenopus* NCoR2 37b+ and 37b− isoforms are readily found in all embryonic and adult tissues analysed [27]. The activity of trans-acting factors [66] in zebrafish, but not *Xenopus*, embryos may prevent splicing from the external site either directly, or indirectly by promoting the use of the internal site. This is supported by the observation that a zebrafish exon 37 minigene introduced into *Xenopus* embryos gave approximately equal amounts of 37b+ and 37b− transcripts. The final outcome of alternative splicing, however, is likely to depend on a combination of the intrinsic strength of the splice sites, determined by nucleotide sequence, and the activity of a number of trans-acting factors.

Analyses of differences in alternative splicing patterns between humans and mice have largely come to a different conclusion. Using transgenic mice that contain part of human chromosome 21, and looking at genes whose splicing patterns differ between mice and humans, Barbosa-Morais *et al.* [67] found that the human genes maintain the human pattern, even in the mouse context, concluding that species-specific patterns of alternative splicing are driven by differences within the genes rather than by changes in the trans-acting factors [67]. The results presented here indicate that differences in the activity of trans-acting factors between species can also play a significant role.

**Figure 6.**
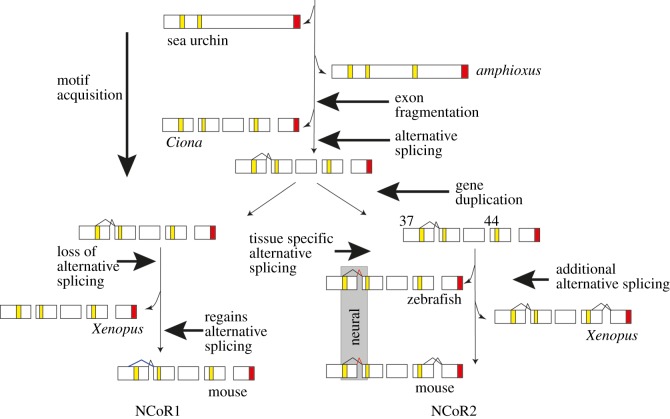
The pathway to diversity for the NCoR family of co-repressors. *Strongylocentrotus purpuratus* (sea urchin) encodes two CoRNR boxes, but this increases to three in the cephalochordate amphioxus. Further motifs, identified by similarity to those in vertebrates, are found in the urochordate *Ciona intestinalis.* An additional CoRNR box motif will increase the range of nuclear receptors to which the co-repressor can bind. While the C-terminal interaction domains are encoded by a single exon in amphioxus they are encoded by at least 10 exons in *Ciona* and the vertebrates (for clarity, not all exons are shown). The ability to restore exon 37b alternative splicing in NCoR1 suggests that alternative splicing of this exon arose in the NCoR-family gene before duplication, which happened during the vertebrate genome duplication event after the divergence of *Ciona* and the vertebrates [69]. Of the two resulting paralogues, NCoR1 lost the alternative splicing of exon 37b by point mutation of the splice donor. Mammals, however, have recovered the capacity for alternative splicing to generate an isoform that lacks the first CoRNR box but which employs a different splice donor (blue lines). The alternative splicing of NCoR2 exon 37 is apparent in the teleost zebrafish, where, like mammals such as the mouse, the presence of the long form (red lines) including CoRNR box1 is tissue-specific, being found only in neural tissue. In addition *Xenopus* and the mouse undergo alternative splicing to generate isoforms of exon 44 [27]. Zebrafish lack the capacity for exon 44 alternative splicing. CoRNR boxes are in yellow and the SHARP domain in red.

A difference between vertebrates and other deuterostomes may lie in the increased complexity of their gene regulatory networks [68]. The vertebrate co-repressors NCoR1 and NCoR2 exemplify this because they interact with an impressively broad range of transcription factors by generating isoforms in which the interaction domains contain different complements of the CoRNR box motifs. In contrast, the sea urchin co-repressor is much simpler with one fewer CoRNR boxes and a lack of carboxy-terminal isoforms. In this paper, we detail the pathway leading to the increased diversity of vertebrate co-repressor isoforms (figure 6), highlighting the role of SLiMs located within IDRs, and their deployment by alternative splicing. We therefore identify a mechanism that generates functional diversity in a transcription-associated protein, a critical contributory factor in determining organismal complexity.

## 4. Material and methods

### 4.1. Sequence alignment

The accession numbers of genes used in the comparisons are listed in the electronic supplementary material, table S1. Where the invertebrate NCoR-family orthologue was not annotated, candidates were identified by BLAST comparisons using vertebrate NCoR-family motifs. Candidates with at least two CoRNR box motifs and a C-terminal SHARP interaction motif in the correct order were further validated by the presence of two upstream SANT domains. The sequence of the protein was then inferred from a combination of manual annotation and reference to online annotation. Multiple sequences were aligned using ClustalW2 and Clustal Omega (EBI-EMBL) using standard criteria.

### 4.2. RT-PCR, cloning and sequencing

Zebrafish (AB mixed with Tubingen) total RNA was isolated from five to 10 embryos at the developmental stages described in the text or at 26–28 hpf using TRI Reagent^®^ (Sigma) and purified using RNeasy Micro Kit (Qiagen). *Xenopus* total RNA was isolated from three to five neurula stage embryos by phenol extraction and precipitation. Alternative splicing was assessed by conversion of RNA into cDNA using Superscript III reverse transcriptase and random nonameric primers, followed by PCR. PCR used species and exon-specific oligonucleotides primers (electronic supplementary material, table S2) and Platinum Taq polymerase (ThermoBioscience) or ReadyMix™ Taq (Sigma). Where described, PCR products were resolved on 1–2% agarose, 1× TBE or 0.5× TAE gels, cloned directly into the vector pCR2.1 (TA cloning, Invitrogen) and sequenced (Source Bioscience).

### 4.3. Cloning of *Xenopus laevis* and zebrafish exon 37 genomic regions

Total nucleic acid was prepared from 50 *X. laevis* tailbud embryos [70] and treated with RNAse. Sets of primers were designed from the *X. laevis* genome assembly v6 on Xenbase [71] to amplify exon 37 of NCoR2 and NCoR1 with approximately 250 base pairs of upstream and downstream intron sequence. The PCR products were cloned into pCR2.1 (Invitrogen, TA cloning) and sequenced (Source Bioscience). The genomic fragments were then blunt-end cloned into the Nde1 site (blunted) of the splicing vector pTBNde1 [56,72]. This vector is based on pBluescript and contains the CMV enhancer driving the expression of human globin and fibronectin exons separated by an intron. The Nde1 site is located centrally within the intron. The orientation of the cloned insert was determined by sequence.

In the zebrafish genome, NCoR2 exon 36 is separated from exon 37 by a short intron of 97 basepairs. We therefore used a primer 249 basepairs upstream of exon 36, spanning a naturally occurring Nde1 site and a reverse primer 474 basepairs downstream of exon 37 that incorporated an Nde1 site. Fragments were cloned into pCR2.1 (Invitrogen, TA cloning) excised with Nde1, cloned into the Nde1 site of splicing vector pTBNde1 and the orientation checked by sequencing.

### 4.4. Site-directed mutagenesis of NCoR1 exon 37

A single base change was introduced into the NCoR1 exon 37 sequence by site-directed mutagenesis of the clone in pCR2.1 using overlapping oligonucleotides carrying the required mutation. Amplification of the mutated sequence used Vent polymerase (New England Biolabs) to limit further mutation and the final construct checked by sequencing. The fragments from pCR2.1 were then blunt-end cloned into pTBNde1 [72] as described above.

### 4.5. Splicing assays

The NCoR1 and NCoR2 exon 37 constructs in pTBNde1 were grown in media and isolated (plasmid midi-prep kit, Machery-Nagel). Approximately 200 pg of plasmid at 20 pg nl^−1^ was injected into each *X. laevis* embryo at the two-cell stage, and the embryos grown to the mid neurula stage (Nieuwkoop and Faber, stage 16) [73]. Zebrafish embryos were injected at the one-cell stage with 200 pg of plasmid at 400 pg nl^−1^, grown overnight at 32°C and collected at stage 27/28 hpf. Total nucleic acid was then extracted [70] and DNA removed by RNAse-free DNAse digestion. The remaining RNA was precipitated, resuspended and converted to cDNA using reverse transcriptase. The splicing status of the transcripts from the clones was assayed by PCR using forward and reverse primers against the human exons [72] or one human and one *Xenopus-* or zebrafish-specific sequence and the products resolved on 1.5–2% agarose gels.

## Supplementary Material

Supplementary Table 1. Supplementary Figure 1. Supplementary Figure 2. Supplementary Figure 3. Supplementary Table 2.

## Data accessibility

Additional datasets supporting this article have been uploaded as part of the electronic supplementary material.

## References

[1] Levine M, Tjian R 2003 Transcription regulation and animal diversity. Nature 424, 147–151. (doi:10.1038/nature01763) 12853946 10.1038/nature01763

[2] Levine M, Cattoglio C, Tjian R 2014 Looping back to leap forward: transcription enters a new era. Cell 157, 13–25. (doi:10.1016/j.cell.2014.02.009) 24679523 10.1016/j.cell.2014.02.009 PMC4059561

[3] Weatheritt RJ, Gibson TJ 2012 Linear motifs: lost in (pre)translation. Trends Biochem. Sci. 37, 333–341. (doi:10.1016/j.tibs.2012.05.001) 22705166 10.1016/j.tibs.2012.05.001

[4] Van Roey K, Uyar B, Weatheritt RJ, Dinkel H, Seiler M, Budd A, Gibson TJ, Davey NE 2014 Short linear motifs: ubiquitous and functionally diverse protein interaction modules directing cell regulation. Chem. Rev. 114, 6733–6778. (doi:10.1021/cr400585q) 24926813 10.1021/cr400585q

[5] Wagner GP, Lynch VJ 2008 The gene regulatory logic of transcription factor evolution. Trends Ecol. Evol. 23, 377–385. (doi:10.1016/j.tree.2008.03.006) 18501470 10.1016/j.tree.2008.03.006

[6] Kelemen O, Convertini P, Zhang Z, Wen Y, Shen M, Falaleeva M, Stamm S 2013 Function of alternative splicing. Gene 514, 1–30. (doi:10.1016/j.gene.2012.07.083) 22909801 10.1016/j.gene.2012.07.083 PMC5632952

[7] Blencowe BJ 2006 Alternative splicing: new insights from global analyses. Cell 126, 37–47. (doi:10.1016/j.cell.2006.06.023) 16839875 10.1016/j.cell.2006.06.023

[8] Boyer LA, Latek RR, Peterson CL 2004 The SANT domain: a unique histone-tail-binding module? *Nat. Rev.* Mol. Cell Biol. 5, 158–163. (doi:10.1038/nrm1314) 10.1038/nrm1314 15040448

[9] Lee SK, Kim JH, Lee YC, Cheong J, Lee JW 2000 Silencing mediator of retinoic acid and thyroid hormone receptors, as a novel transcriptional corepressor molecule of activating protein-1, nuclear factor-κB, and serum response factor. J. Biol. Chem. 275, 12 470–12 474. (doi:10.1074/jbc.275.17.12470) 10.1074/jbc.275.17.12470 10777532

[10] Li J, Wang J, Wang J, Nawaz Z, Liu JM, Qin J, Wong J 2000 Both corepressor proteins SMRT and N-CoR exist in large protein complexes containing HDAC3. EMBO J. 19, 4342–4350. (doi:10.1093/emboj/19.16.4342) 10944117 10.1093/emboj/19.16.4342 PMC302030

[11] Xu L, Glass CK, Rosenfeld MG 1999 Coactivator and corepressor complexes in nuclear receptor function. Curr. Opin. Genet. Dev. 9, 140–147. (doi:10.1016/S0959-437X(99)80021-5) 10322133 10.1016/S0959-437X(99)80021-5

[12] Yu J, Li Y, Ishizuka T, Guenther MG, Lazar MA 2003 A SANT motif in the SMRT corepressor interprets the histone code and promotes histone deacetylation. EMBO J. 22, 3403–3410. (doi:10.1093/emboj/cdg326) 12840002 10.1093/emboj/cdg326 PMC165650

[13] Guenther MG, Barak O, Lazar MA 2001 The SMRT and N-CoR corepressors are activating cofactors for histone deacetylase 3. Mol. Cell Biol. 21, 6091–6101. (doi:10.1128/MCB.21.18.6091-6101.2001) 11509652 10.1128/MCB.21.18.6091-6101.2001 PMC87326

[14] Hu X, Lazar MA 1999 The CoRNR motif controls the recruitment of corepressors by nuclear hormone receptors. Nature 402, 93–96. (doi:10.1038/47069) 10573424 10.1038/47069

[15] Hu X, Li Y, Lazar MA 2001 Determinants of CoRNR-dependent repression complex assembly on nuclear hormone receptors. Mol. Cell Biol. 21, 1747–1758. (doi:10.1128/MCB.21.5.1747-1758.2001) 11238912 10.1128/MCB.21.5.1747-1758.2001 PMC86726

[16] Cohen RN, Brzostek S, Kim B, Chorev M, Wondisford FE, Hollenberg AN 2001 The specificity of interactions between nuclear hormone receptors and corepressors is mediated by distinct amino acid sequences within the interacting domains. Mol. Endocrinol. 15, 1049–1061. (doi:10.1210/mend.15.7.0669) 11435607 10.1210/mend.15.7.0669

[17] Makowski A, Brzostek S, Cohen RN, Hollenberg AN 2003 Determination of nuclear receptor corepressor interactions with the thyroid hormone receptor. Mol. Endocrinol. 17, 273–286. (doi:10.1210/me.2002-0310) 12554754 10.1210/me.2002-0310

[18] Watson PJ, Fairall L, Schwabe JW 2012 Nuclear hormone receptor co-repressors: structure and function. Mol. Cell Endocrinol. 348, 440–449. (doi:10.1016/j.mce.2011.08.033) 21925568 10.1016/j.mce.2011.08.033 PMC3315023

[19] Beato M 1991 Transcriptional control by nuclear receptors. FASEB J. 5, 2044–2051. 2010057 10.1096/fasebj.5.7.2010057

[20] Mangelsdorf DJ et al. 1995 The nuclear receptor superfamily: the second decade. Cell 83, 835–839. (doi:10.1016/0092-8674(95)90199-X) 8521507 10.1016/0092-8674(95)90199-x PMC6159888

[21] Rastinejad F, Wagner T, Zhao Q, Khorasanizadeh S 2000 Structure of the RXR-RAR DNA-binding complex on the retinoic acid response element DR1. EMBO J. 19, 1045–1054. (doi:10.1093/emboj/19.5.1045) 10698945 10.1093/emboj/19.5.1045 PMC305643

[22] Jepsen K, Rosenfeld MG 2002 Biological roles and mechanistic actions of co-repressor complexes. J. Cell Sci. 115, 689–698. 11865025 10.1242/jcs.115.4.689

[23] Downes M, Burke LJ, Bailey PJ, Muscat GE 1996 Two receptor interaction domains in the corepressor, N-CoR/RIP13, are required for an efficient interaction with Rev-erbAα and RVR: physical association is dependent on the E region of the orphan receptors. Nucleic Acids Res. 24, 4379–4386. (doi:10.1093/nar/24.22.4379) 8948627 10.1093/nar/24.22.4379 PMC146280

[24] Malartre M, Short S, Sharpe C 2006 *Xenopus* embryos lacking specific isoforms of the corepressor SMRT develop abnormal heads. Dev. Biol. 292, 333–343. (doi:10.1016/j.ydbio.2006.01.007) 16500640 10.1016/j.ydbio.2006.01.007

[25] Faist F, Short S, Kneale G, Sharpe CR 2009 Alternative splicing determines the interaction of SMRT isoforms with nuclear receptor–DNA complexes. Biosci. Rep. 29, 143–149. (doi:10.1042/BSR20080093) 18752469 10.1042/BSR20080093

[26] Malartre M, Short S, Sharpe C 2004 Alternative splicing generates multiple SMRT transcripts encoding conserved repressor domains linked to variable transcription factor interaction domains. Nucleic Acids Res. 32, 4676–4686. (doi:10.1093/nar/gkh786) 15342788 10.1093/nar/gkh786 PMC516058

[27] Short S, Malartre M, Sharpe C 2005 SMRT has tissue-specific isoform profiles that include a form containing one CoRNR box. Biochem. Biophys. Res. Commun. 334, 845–852. (doi:10.1016/j.bbrc.2005.06.175) 16026760 10.1016/j.bbrc.2005.06.175

[28] Goodson M, Jonas BA, Privalsky MA 2005 Corepressors: custom tailoring and alterations while you wait. Nucl. Recept. Signal. 3, e003 (doi:10.1621/nrs.03003) 16604171 10.1621/nrs.03003 PMC1402215

[29] Goodson ML, Jonas BA, Privalsky ML 2005 Alternative mRNA splicing of SMRT creates functional diversity by generating corepressor isoforms with different affinities for different nuclear receptors. J. Biol. Chem. 280, 7493–7503. (doi:10.1074/jbc.M411514200) 15632172 10.1074/jbc.M411514200 PMC2720035

[30] Mengeling BJ, Goodson ML, Bourguet W, Privalsky ML 2012 SMRTε, a corepressor variant, interacts with a restricted subset of nuclear receptors, including the retinoic acid receptors α and β. Mol. Cell Endocrinol. 351, 306–316. (doi:10.1016/j.mce.2012.01.006) 22266197 10.1016/j.mce.2012.01.006 PMC3288673

[31] Goodson ML, Mengeling BJ, Jonas BA, Privalsky ML 2011 Alternative mRNA splicing of corepressors generates variants that play opposing roles in adipocyte differentiation. J. Biol. Chem. 286, 44 988–44 999. (doi:10.1074/jbc.M111.291625) 10.1074/jbc.M111.291625 PMC3247966 22065574

[32] Heinzel T et al. 1997 A complex containing N-CoR, mSin3 and histone deacetylase mediates transcriptional repression. Nature 387, 43–48. (doi:10.1038/387043a0) 9139820 10.1038/387043a0

[33] Nagy L, Kao H-Y, Chakravarti D, Lin RJ, Hassig CA, Ayer DE, Schreiber SL, Evans RM 1997 Nuclear receptor repression mediated by a complex containing SMRT, mSin3A, and histone deacetylase. Cell 89, 373–380. (doi:10.1016/S0092-8674(00)80218-4) 9150137 10.1016/s0092-8674(00)80218-4

[34] Jepsen K et al. 2000 Combinatorial roles of the nuclear receptor corepressor in transcription and development. Cell 102, 753–763. (doi:10.1016/S0092-8674(00)00064-7) 11030619 10.1016/s0092-8674(00)00064-7

[35] Jepsen K, Solum D, Zhou T, McEvilly RJ, Kim HJ, Glass CK, Hermanson O, Rosenfeld MG 2007 SMRT-mediated repression of an H3K27 demethylase in progression from neural stem cell to neuron. Nature 450, 415–419. (doi:10.1038/nature06270) 17928865 10.1038/nature06270

[36] Yang J, Tang Y, Liu H, Guo F, Ni J, Le W 2014 Suppression of histone deacetylation promotes the differentiation of human pluripotent stem cells towards neural progenitor cells. BMC Biol. 12, 95 (doi:10.1186/s12915-014-0095-z) 25406762 10.1186/s12915-014-0095-z PMC4254204

[37] Weston AD, Blumberg B, Underhill TM 2003 Active repression by unliganded retinoid receptors in development: less is sometimes more. J. Cell Biol. 161, 223–228. (doi:10.1083/jcb.200211117) 12719467 10.1083/jcb.200211117 PMC2172895

[38] Castelo-Branco G et al. 2014 Neural stem cell differentiation is dictated by distinct actions of nuclear receptor corepressors and histone deacetylases. Stem Cell Rep. 3, 502–515. (doi:10.1016/j.stemcr.2014.07.008) 10.1016/j.stemcr.2014.07.008 PMC4266002 25241747

[39] Mottis A, Mouchiroud L, Auwerx J 2013 Emerging roles of the corepressors NCoR1 and SMRT in homeostasis. Genes Dev. 27, 819–835. (doi:10.1101/gad.214023.113) 23630073 10.1101/gad.214023.113 PMC3650221

[40] Reilly SM et al. 2010 Nuclear receptor corepressor SMRT regulates mitochondrial oxidative metabolism and mediates aging-related metabolic deterioration. Cell Metab. 12, 643–653. (doi:10.1016/j.cmet.2010.11.007) 21109196 10.1016/j.cmet.2010.11.007 PMC3033658

[41] Wei Y, Ma D, Gao Y, Zhang C, Wang L, Liu F 2014 Ncor2 is required for hematopoietic stem cell emergence by inhibiting Fos signaling in zebrafish. Blood 124, 1578–1585. (doi:10.1182/blood-2013-11-541391) 25006126 10.1182/blood-2013-11-541391

[42] He LZ, Guidez F, Tribioli C, Peruzzi D, Ruthardt M, Zelent A, Pandolfi PP 1998 Distinct interactions of PML-RARα and PLZF-RARα with co-repressors determine differential responses to RA in APL. Nat. Genet. 18, 126–135. (doi:10.1038/ng0298-126) 9462740 10.1038/ng0298-126

[43] Lin RJ, Nagy L, Inoue S, Shao W, Miller WH, Evans RM 1998 Role of the histone deacetylase complex in acute promyelocytic leukaemia. Nature 391, 811–814. (doi:10.1038/35895) 9486654 10.1038/35895

[44] Mengeling BJ, Phan TQ, Goodson ML, Privalsky ML 2011 Aberrant corepressor interactions implicated in PML-RARα and PLZF-RARα leukemogenesis reflect an altered recruitment and release of specific NCoR and SMRT splice variants. J. Biol. Chem. 286, 4236–4247. (doi:10.1074/jbc.M110.200964) 21131350 10.1074/jbc.M110.200964 PMC3039402

[45] Hong SH, Dvorak-Ewell M, Stevens HY, Barish GD, Castro GL, Nofsinger R, Frangos JA, Shoback D, Evans RM 2013 Rescue of a primary myelofibrosis model by retinoid-antagonist therapy. Proc. Natl Acad. Sci. USA 110, 18 820–18 825. (doi:10.1073/pnas.1318974110) 10.1073/pnas.1318974110 PMC3839709 24191050

[46] Alrfaei BM, Vemuganti R, Kuo JS 2013 microRNA-100 targets SMRT/NCOR2, reduces proliferation, and improves survival in glioblastoma animal models. PLoS ONE 8, e80865 (doi:10.1371/journal.pone.0080865) 24244722 10.1371/journal.pone.0080865 PMC3828259

[47] Nofsinger RR et al. 2008 SMRT repression of nuclear receptors controls the adipogenic set point and metabolic homeostasis. Proc. Natl Acad. Sci. USA 105, 20 021–20 026. (doi:10.1073/pnas.0811012105) 19066220 10.1073/pnas.0811012105 PMC2598729

[48] Romero PR et al. 2006 Alternative splicing in concert with protein intrinsic disorder enables increased functional diversity in multicellular organisms. Proc. Natl Acad. Sci. USA 103, 8390–8395. (doi:10.1073/pnas.0507916103) 16717195 10.1073/pnas.0507916103 PMC1482503

[49] Shi Y, Downes M, Xie W, Kao H-Y, Ordentlich P, Tsai C-C, Hon M, Evans RM 2001 Sharp, an inducible cofactor that integrates nuclear receptor repression and activation. Genes Dev. 15, 1140–1151. (doi:10.1101/gad.871201) 11331609 10.1101/gad.871201 PMC312688

[50] Perissi V et al. 1999 Molecular determinants of nuclear receptor-corepressor interaction. Genes Dev. 13, 3198–3208. (doi:10.1101/gad.13.24.3198) 10617569 10.1101/gad.13.24.3198 PMC317209

[51] Nagy L et al. 1999 Mechanism of corepressor binding and release from nuclear hormone receptors. Genes Dev. 13, 3209–3216. (doi:10.1101/gad.13.24.3209) 10617570 10.1101/gad.13.24.3209 PMC317208

[52] Lohr U, Pick L 2005 Cofactor-interaction motifs and the cooption of a homeotic Hox protein into the segmentation pathway of *Drosophila melanogaster*. Curr. Biol. 15, 643–649. (doi:10.1016/j.cub.2005.07.022) 15823536 10.1016/j.cub.2005.02.048

[53] Su Z, Wang J, Yu J, Huang X, Gu X 2006 Evolution of alternative splicing after gene duplication. Genome Res. 16, 182–189. (doi:10.1101/gr.4197006) 16365379 10.1101/gr.4197006 PMC1361713

[54] Horowitz DS, Krainer AR 1994 Mechanisms for selecting 5′ splice sites in mammalian pre-mRNA splicing. Trends Genet. 10, 100–106. (doi:10.1016/0168-9525(94)90233-X) 8178363 10.1016/0168-9525(94)90233-x

[55] Yeo G, Burge CB 2004 Maximum entropy modeling of short sequence motifs with applications to RNA splicing signals. J. Comput. Biol. 11, 377–394. (doi:10.1089/1066527041410418) 15285897 10.1089/1066527041410418

[56] Baralle M, Baralle D, De Conti L, Mattocks C, Whittaker J, Knezevich A, ffrench-Constant C, Baralle FE 2003 Identification of a mutation that perturbs *NF1* agene splicing using genomic DNA samples and a minigene assay. J. Med. Genet. 40, 220–222. (doi:10.1136/jmg.40.3.220) 12624144 10.1136/jmg.40.3.220 PMC1735390

[57] Varlakhanova N, Hahm JB, Privalsky ML 2011 Regulation of SMRT corepressor dimerization and composition by MAP kinase phosphorylation. Mol. Cell Endocrinol. 332, 180–188. (doi:10.1016/j.mce.2010.10.010) 20965228 10.1016/j.mce.2010.10.010 PMC3011023

[58] Ryo A, Wulf G, Lee TH, Lu KP 2009 Pinning down HER2-ER crosstalk in SMRT regulation. Trends Biochem. Sci. 34, 162–165. (doi:10.1016/j.tibs.2008.12.004) 19269830 10.1016/j.tibs.2008.12.004

[59] Jonas BA, Privalsky ML 2004 SMRT and N-CoR corepressors are regulated by distinct kinase signaling pathways. J. Biol. Chem. 279, 54 676–54 686. (doi:10.1074/jbc.M410128200) 10.1074/jbc.M410128200 PMC2653424 15491994

[60] Wray GA 2007 The evolutionary significance of *cis*-regulatory mutations. Nat. Rev. Genet. 8, 206–216. (doi:10.1038/nrg2063) 17304246 10.1038/nrg2063

[61] Delsuc F, Tsagkogeorga G, Lartillot N, Philippe H 2008 Additional molecular support for the new chordate phylogeny. Genesis 46, 592–604. (doi:10.1002/dvg.20450) 19003928 10.1002/dvg.20450

[62] Gissi C, Pesole G, Cattaneo E, Tartari M 2006 Huntingtin gene evolution in Chordata and its peculiar features in the ascidian *Ciona* genus. BMC Genomics 7, 288 (doi:10.1186/1471-2164-7-288) 17092333 10.1186/1471-2164-7-288 PMC1636649

[63] He X, Zhang J 2005 Rapid subfunctionalization accompanied by prolonged and substantial neofunctionalization in duplicate gene evolution. Genetics 169, 1157–1164. (doi:10.1534/genetics.104.037051) 15654095 10.1534/genetics.104.037051 PMC1449125

[64] Conant GC, Wolfe KH 2008 Turning a hobby into a job: how duplicated genes find new functions. Nat. Rev. Genet. 9, 938–950. (doi:10.1038/nrg2482) 19015656 10.1038/nrg2482

[65] Buljan M, Chalancon G, Dunker AK, Bateman A, Balaji S, Fuxreiter M, Babu MM 2013 Alternative splicing of intrinsically disordered regions and rewiring of protein interactions. Curr. Opin. Struct. Biol. 23, 443–450. (doi:10.1016/j.sbi.2013.03.006) 23706950 10.1016/j.sbi.2013.03.006

[66] De Conti L, Baralle M, Buratti E 2013 Exon and intron definition in pre-mRNA splicing. Wiley Interdiscip. Rev. RNA 4, 49–60. (doi:10.1002/wrna.1140) 23044818 10.1002/wrna.1140

[67] Barbosa-Morais NL et al. 2012 The evolutionary landscape of alternative splicing in vertebrate species. Science 338, 1587–1593. (doi:10.1126/science.1230612) 23258890 10.1126/science.1230612

[68] Cheatle Jarvela AM, Hinman VF 2015 Evolution of transcription factor function as a mechanism for changing metazoan developmental gene regulatory networks. EvoDevo 6, 3 (doi:10.1186/2041-9139-6-3) 25685316 10.1186/2041-9139-6-3 PMC4327956

[69] Dehal P, Boore JL 2005 Two rounds of whole genome duplication in the ancestral vertebrate. PLoS Biol. 3, e314 (doi:10.1371/journal.pbio.0030314) 16128622 10.1371/journal.pbio.0030314 PMC1197285

[70] Zeng Z, Sharpe CR, Simons JP, Gorecki DC 2006 The expression and alternative splicing of alpha-neurexins during *Xenopus* development. Int. J. Dev. Biol. 50, 39–46. (doi:10.1387/ijdb.052068zz) 16323076 10.1387/ijdb.052068zz

[71] Bowes JB, Snyder KA, Segerdell E, Jarabek CJ, Azam K, Zorn AM, Vize PD 2010 Xenbase: gene expression and improved integration. Nucleic Acids Res. 38, D607–D612. (doi:10.1093/nar/gkp953) 19884130 10.1093/nar/gkp953 PMC2808955

[72] Pagani F, Stuani C, Romano M, Zuccato E, Niksic M, Giglio L, Faraguna D, Baralle FE 2000 Splicing factors induce cystic fibrosis transmembrane regulator exon 9 skipping through a nonevolutionary conserved intronic element. J. Biol. Chem. 275, 21 041–21 047. (doi:10.1074/jbc.M910165199) 10.1074/jbc.M910165199 10766763

[73] Nieuwkoop PD, Faber J 1967 Normal table of *Xenopus laevis*. North Holland: Amsterdam.

