# Supplementary material for: Short linear motif acquisition, exon formation and alternative splicing determine a pathway to diversity for NCoR-family co-repressors
Source: Open Biol. 2015 Aug 19;5(8):150063. doi: 10.1098/rsob.150063 (PMC4554918; doi:10.1098/rsob.150063)
Supplement: Supplementary Table 1. Supplementary Figure 1. Supplementary Figure 2. Supplementary Figure 3. Supplementary Table 2. [file rsob150063supp1.pdf]

Supplementary information

Supplementary Table 1
Genes and species

Supplementary Figure 1
Alignment of selected tetrapod NCoR1and NCoR2 C-terminal sequences

Supplementary Figure 2
Alignment of NCoR-family motifs for human, Xenopus, sea-squirt, amphioxus and sea urchin shows the progressive acquisition of motifs

Supplementary Figure 3
The unusual arrangement of Medaka exon 37.

Supplementary Table 2
Oligonucleotides used in cloning and analysis

Supplementary information

Supplementary Table 1

Species	Gene	Identity	Evidence	Notes
Sea Urchin *Strongylocentrotus purpuratus*	NCoR-family	Echinobase SPU_003509 Sp-NCoR2	- predicted gene<br>- RNAseq transcriptome: WHL22.467600.1	• confirmed by reference to motifs
Acorn Worm *Sarcoglossus kowalevskii*	NCoR-family	Metazome v3.0 scaffold_505: 392433-398065	Sakowv30030073m Sakowv30030074m	• confirmed by reference to motifs and to PASA aligned ESTs
Lancelet (Amphioxus) *Branchiostoma floridae*	NCoR-family	fgenesh_2pgscaffold_214000029<br>XM_002611906	- predicted gene	• confirmed by reference to motifs
Sea Squirt *Ciona intestinalis*	NCoR-family	XM_009861727	- predicted gene<br>- ESTs	• confirmed by reference to motifs
Lamprey *Petromyzon maritimus*	NCoRA (NCoR1)	In two linked scaffold annotations<br>i Scaffold 478495 63 – 19247 exon 37-43 ENSPMAG00000000795<br><br>ii Scaffold 478495 29066 – 34712 exon 44-47 ENSPMAG00000000800	-predicted gene<br><br>-predicted gene	• confirmed by reference to motifs<br><br>• identified by confirmed to motifs Not all exons annotated
Lamprey *Petromyzon maritimus*	NCoR-B (NCoR2)	Scaffold GL478817 1503-38391 exon 37-44 ENSPMAG00000005368	- predicted gene<br>- de novo sequencing	• predicted as NCoR2-<br>•exons 37-40 annotated by de-novo cDNA sequencing and comparison to genome sequence.<br>• gene sequence incomplete after exon 44.
Lamprey *Lethenteron japonicum*	NCoR-A (NCoR1)	Scaffold 00020<br>KE993691	- predicted gene	• confirmed by reference to motifs.<br>• incomplete annotation due to lack of EST data
Lamprey *Lethenteron japonicum*	NCoR-B (NCoR2)	Scaffold 00155<br>KE993826	-predicted gene	• confirmed by reference to motifs.<br>• incomplete annotation due to lack of EST data
Spiny dogfish *Squalus acanthus*	NCoR2	ESTs<br>EB158249<br>EB158250<br>DV500562	- predicted gene	• predicted similar to chick NCoR2<br>• sequence incomplete
Little skate *Leucoraja erinacea*	NCoR2	EST<br>ctg89959 (skatebase.org)	- predicted gene	• confirmed by reference to motifs.<br>• sequence incomplete
Zebrafish	NCoR1	Chromosome 5: 43496834-	- predicted gene	• used transcript

Danio rerio		43615097<br>ENSDARG00000035285	- manual annotation by Havana	ENSDART000000 97574
Zebrafish<br>*Danio rerio*	NCoR2	Chromosome 8: 45419869-45564976<br>ENSDARG00000000966	- predicted gene<br>- manual annotation by Havana	• predicted inclusion of exon 37b in ENSDART000000 01092
Medaka<br>*Oryzias latipes*	NCoR1	Chromosome14: 22287614-22358117<br>ENSORLG00000011992	- predicted gene	• used transcript ENSORLT000000 15018
Medaka<br>*Oryzias latipes*	NCoR2	Chromosome 9: 20215207-20306020<br>ENSORLG00000014008	- predicted gene	• used transcript ENSORLT000000 17560<br>contains stop codon in prospective exon 37b
Clawed toad<br>*Xenopus tropicalis*	NCoR1	Xenbase XB-GENE-483288<br>Scaffold_2:30433079-30534333 v7.1	- predicted gene<br>- manual annotation	Refseq<br>NM_001032342.1
Clawed toad<br>*Xenopus laevis*	NCoR2	Xenbase XB-GENE-5963159<br>Scaffold_141:267380-323271	- predicted gene<br>- manual annotation<br>- comparison to cDNA	RefSeq<br>NM_001091023.1<br>Includes exon 37b
Chicken<br>*Gallus gallus*	NCoR1	Chromosome 19 Gene ID 417598<br>In NW_003764121	- predicted gene but not annotated on ENSEMBL	• used transcript XM_004946661
Chicken<br>*Gallus gallus*	NCoR2/SMRT	Chromosome 15: 4589898-4719390	- predicted gene	• used transcript ENSGALT000000 04933<br>lacks 37b, but added from genome sequence.
Mouse<br>*Mus musculus*	NCoR1	Chromosome 11: 62316426-62458541<br>ENSMUSG00000018501	- predicted gene<br>- manual annotation	• used transcript ENSMUST000000 18645
Mouse<br>*Mus musculus*	NCoR2/SMRT	Chromosome 5: 125017153-125179219<br>ENSMUSG00000029478	- predicted gene<br>- manual annotation	• used transcript ENSMUST000001 11393<br>lacks 37b, but added from genome sequence and validated by RT-PCR (22)
Dog<br>*Canis familiaris*	NCoR1	Chromosome 5: 39707813-39836089<br>ENSCAFG00000018040	- predicted gene	•used transcript ENSCAFT000000 28704
Dog<br>*Canis familiaris*	NCoR2/SMRT	Chromosome 26: 5315660-5493741<br>ENSCAFG00000006985	- predicted gene	• used transcript ENSCAFT000000 11198<br>lacks 37b, but added from genome sequence.
Human<br>*Homo sapiens*	NCoR1	Chromosome 17: 15932471-16121499<br>ENSG00000141027	- predicted gene<br>- manual annotation by Havana	• used transcript ENST0000026871 2
Human<br>*Homo sapiens*	NCoR2/SMRT	Chromosome 12: 124808961-125052135<br>ENSG00000196498	- predicted gene<br>- manual annotation by Havana	•used transcript ENST0000040520 1<br>Validated by AI762677 and RT-PCR (22)

Supplementary figure 1

Alignment of selected tetrapod NCoR1 and NCoR2 C-terminal sequences

Clustal alignment of human (hu), dog (ca), mouse (mu), chick (gg) and Xenopus tropicalis (xt) or Xenopus laevis (xl) NCoR1 (NC) and NCoR2 (SM) protein sequence across the region encoded from exon 37 to the end of the protein encoded in exon 46. Alternating exons in black and blue, residues encoded by triplets that span an exon boundary are in red. Motifs are in shaded blocks. Conserved motifs were deemed to have greater than 50% identity over 12 or more residues, bounded by conserved residues, or 7 or more contiguous identical residues with no gap bigger than 4 when all sequences are considered.

```
huNC             -----MPLPAGGPSISQGLPASRYNTAADALAALVDAAASAPQMDVSKTKESKHEAARLE 55
caNC             -----MPLPAGGPSISQGLPASRYSTAADALAALVDAAASAPQMEVSKTKESKHEAARLE 55
muNC             -----MPLPSGGPSISQGLPASRYNTAADALAALVDAAASAPQMDVSKTKESKHEAARLE 55
ggNC             -----MQLTPGAPSITQGLPASRYNTAADALAALVDAAASAPQMEVAKGKDTKHEGTRIE 55
xtNC             -----MPPTPGAASITQGIPASRYSTAADALAALVDAAASAPQMEVVKPKEMKHDPARSE 55
huSM            RS-TSTSSPVRPAATFPPATHCPLGGTLDGVYPTLMEPVLLPKEAP--RVARPERPRADTG 58
caSM            RS-TSTSSPVRPAATFPPTTHCPLGGTLDGVYPTLMEPVLPPKEAP--RVARPERPRADAG 58
muSM            RS-TSTSSPVRPAATFPPATHCPLGGTLEGVYPTLMEPVLLPKETS--RVARPERPRVDAG 58
ggSM            RSSTTTTSPIRSSAFPSASALRSSIGAAD-GYAAMLDPAVLQKEASRAREAKAERAQTDSS 59
xlSM            RT-SSSSSPIRSSMQLTAGSHR-SVSGSE-GYQS-ADHVMLQKEMIRTRDSKLERPRTEN- 56
                            .   .        .       :  : .    :    :    ::  .

huNC             ENLRSRSAAVSEQQQLEQKTLEVEKRSVQCLYTSSAFPSGKPQPHSS------------V 103
caNC             ENLRSRSAAVSEQQQLEQKTLEVEKRSVQCLYTSAAFPSGKPQPHSS------------V 103
muNC             ENLRSRSAAVSEQQQLEQKNLEVEKRSVQCVCTSSALPSGKAQPHAS------------V 103
ggNC             ENVGRRSAVVTEQQQMEQKTLEVEKRAVQCPYTSANFSGGKSQGQSST-----------V 104
xtNC             ESLSRR--NVLEQQQQQQQ-IDCERRVMQSPYTSSSFSGSKSQGQPSP-----------A 101
huSM             HAFLAKPPARSGLEPASSPSKGSEPRPLVPPVSGHATIARTPAKNLAPHHASPDPPAPPA 118
caSM             HAFLAKPQARVGLEPASPPSKGSEPRPLAPPGSSHSAIARTPAKSLAPHHAGPDQPAPPA 118
muSM             HAFLTKPPAR---EPASSPSKSSEPRSLAPPSSSHTAIARTPAKNLAPHHASPDPPAP-T 114
ggSM             -VFTSKLPSGASLEQSPSPIKAMESRPLPASGPGSSHHYSRGQGKSQQHHHPLDQQHAAS 118
xlSM             -FYNSKLPT-VAIEQT-SPIKAVESRPLTSTGAGSAHHYNNGQGKSQQ-HQPLDQS-TVS 111
                      :       :         * * :    ..

                                    Motif 1
huNC             VYSEAGKDKGP-PPKSRYEEELRTRGKTTITAANFIDVIITRQIASDKDARERGSQSSDS 162
caNC             VYSEAGKDKGP-PPKSRYEEELRTRGKTTITAANFIDVIITRQIASDKDARERGSQSSDS 162
muNC             VYSEAGKDKGP-PPKSRYEEELRTRGKTTITAANFIDVIITRQIASDKDARERGSQSSDS 162
ggNC             VYSEAGKEKGP-PPKSRYEEELRTRGKTTITAANFIDVIITRQIASDKDARDRGSQSSDS 163
xtNC             VYSEAGKEKTA-HTKSRYVEELRMRGKTTITAANFIDVIITQQIASDKDGRDRNSQSSDS 160
huSM             SASDPHREKTQSKPFSIQELELRSLGKTTLTAATFIDAIIMRQIAHDKGAREGGALANGS 178
caSM             STTDLHREKTQSKPFSIQELELRSLGKTTLTAATFIDAIIMRQIAHDKGPREGGSLANDS 178
muSM             SASDLHREKTQSKPFSIQELELRSLGKTTLTAATFIDAIITRQIAHERGPREGGSLANDS 174
ggSM             GPEQHSREKSQSKPFSMQEQELRALGKTTTTAASFIDVIIMRGISCDKGQRERGSLSNSS 178
xlSM             AIEQHNRDKTPIKPFSMQEQELRSLGKTTMTAANFIDAIIMRQISCDNGKRERGSLNIDA 171
                    :  ::*    . *    ***  **** ***.***.** : *: :.. *: .:   .:

                 37  38                                      38    39
huNC             SSSLSSHRYETPSDAIEVISPASSPAPPQEKLQTYQPEVVKANQAENDPTRQYEGPLHHY 222
caNC             SSSLSSHRYEAPGDAIEVISPASSPVPPPEKLQAYPPEVVKANQAESEASRQYEGPLHHY 222
muNC             SSSLSSHRYETASDAIEVISPASSPAPPQEKPQAYQPDMVKANQAENESTRQYEGPLHHY 222
ggNC             SSSLSSHRYEAPGDAIEVISPANSPIPTQEKLQPYQQETPKPSQAESDPNRQYEGPIHRY 223
xtNC             SSSHSSHRYDAPRDTIEVISPANSPVQEKE---SYPPEIPKSSQTESESSRKYEGQPNRY 217
huSM             PRDGYHGSS-YSPEGVEPVSPVSSPSLTHDKGLPKHLEELDKSHLEGELRPKQPGPVKL- 236
caSM             PRDGYHGGS-YSPDGVEPISPVSSPSLTHDKGLPKHLEELDKSHLEGDLRHKQPGPGKL- 236
muSM             PGDGYHSGAGYSPDGVEPISPVSSPSLTHDKGLSKPLEELEKSHLEGELRHKQPGPMKL- 233
ggSM             TSDGFHSGY--SPDRIEAVSPVSSPSLSHEKGAK-LLQDVEKGHGEHDPRQKQQASLKAS 235
xlSM             NSDGFPAGY--SPDRLDSISPRNSPGLCHDKRILGATQDLESSGLDQEPRQKQ-GPIKLS 228
                   .        . : :: :** .**    :       :  . .  : :   :  .  :
```

```
                                            Motif 2 39  40
huNC      RPQQESP-SPQQQ----LPPSSQAEGMGQVPRTHRLITLADHICQIITQDFARNQVSSQT 277
caNC      RPQQEPL-SPQQQ----LPASSQADGVAQVPRTHRLITLADHICQIITQDFARNQVSSQP 277
muNC      RSQQESP-SPQQQPP--LPPSSQSEGMGQVPRTHRLITLADHICQIITQDFARNQVPSQ- 278
ggNC      RAQQEPPPSPQQP----PPPSSQAEGMAHVPRTHRLITLADHICQIITQDFARNQAASQA 279
xtNC      RQQQESP-SPQQT------------IPGHVPQTHRLITLADHICQIITQDFARNQPVNQA 264
huSM      GGEAAHLPHLRPLPESQPSSSPLLQTAPGVKGHQRVVTLAQHISEVITQDYTRHHPQQLS 296
caSM      SGEAAHLSHLRPLPESQSSSSPLLQTTPGVKGHQRVVTLAQHISEVITQDYTRHHPQQLN 296
muSM      SAEAAHLPHLRPLPESQPSSSPLLQTAPGIKGHQRVVTLAQHISEVITQDYTRHHPQQLS 293
ggSM      GPEAAHLQHLRQPDGPQPQCQPLQSSQSVKGMNQRVVTLAQHISEVITQDYTRHHPQQLN 295
xlSM      SSEG-HMR--LKAEGQQSPCH----SQPPYGKSQRVVTLAQHISEVITQDYTRHHPQQLH 281
            :                              :*::***:**.::****::*::  .

huNC      PQQPPTSTFQNSPSALVSTPVRTKTSNRYSPESQAQSVHHQ--RPGSRVSPENLVDKSRG 335
caNC      PQQPPTSTFQNSPSALVSTPVRTKTSNRYSPESQSQSVHHQ--RPGSRVSPENLVDKSRG 335
muNC      ---PSTSTFQTSPSALSSTPVRTKTSSRYSPESQSQTVLHP--RPGPRVSPENLVDKSRG 333
ggNC      SLQPPTTTFQNSTPAPTPVSTRAKTSNRYSPEAQSQPVHHQ--RPGARVSPENLSDKSRG 337
xtNC      LQQPPASTFQSTN--PSSTPVRTKASSRFSPESQVQPVHNQ--RPASRVSPENVLDRPRG 320
huSM      APLPAPLYSFPGASCPVLDLRRPPSDLYLPPPDHGAPARGSPHSEGGKRSPEPNKTSVLG 356
caSM      APLPAPLYSFPGASCPVLDLRRPPSDLYLPPPDHGAPARGSPHSEGGKRSPEPSKTSALG 356
muSM      GPLPAPLYSFPGASCPVLDLRRPPSDLYLPPPDHGTPARGSPHSEGGKRSPEPSKTSVLG 353
ggSM      SHIQTPVYSFPGATCPVLDLRRTPSEAYLQQQEHAVASRISPQNEGGKRSPEQSKASAGG 355
xlSM      PQMPASMY-YTGAGCPVLDLRQNPSESVLQHQELAQAARVSPGSNGEKRSPDQNKASALS 340
              ..               :  :.          .        . : **:       .

           41                                             41  42
huNC      S-RPGKSPERSHVSSEPYEPISPPQ--VPVVHEKQDSLLLLSQRGAEPAEQRNDARSPGS 392
caNC      SIRPGKSPERSHVPSESYEPISPPQ--VPVVHEKQDSMLLLSQRGAEPAEQRNDSRSPGS 393
muNC      S-RPGKSPERSHIPSEPYEPISPPQ--GPAVHEKQDSMLLLSQRGVDPAEQRSDSRSPGS 390
ggNC      S-RPGKSPERSHVSSEPYEPISPPQ--VPVVHEKQENVLLLSQR-AEPTEQRTDSRSPGS 393
xtNC      R--PGKSPDRGHIS-EPYEPISPPQ--APLLHAKQDSMLLLSQR-QEPPEQRNDSRSPGN 374
huSM      G------------GEDGIEPVSPPEGMTEPGHSRSAVYPLLYRDGEQTEPSRMGSKSPGN 404
caSM      S------------SEDAIEPVSPPEGLAEPGHPRSTMYPLLYRDGEQAETSRMGSKSPGN 404
muSM      S------------SEDAIEPVSPPEGMTEPGHARSTAYPLLYRDGEQGEP-RMGSKSPGN 400
ggSM      G------------SDNCIEPVSPPDGVGEAEHAKSATYPILYREGEQLDQ-RMGSKSPGN 402
xlSM      G------------SEDGMDPISPPEGMGEPDYSRSTTYPTLYREGEQTEP-RMGSKSPGN 387
                         :  :*:***:      : :.     * :   :    * .::***.

              Motif 3                          42  43 Motif 4    43   44
huNC      ISYLPSFFTKLEN-TSPMVKSKKQEIFRKLNSSGGGDSDMAAAQPGTEIFNLPAVTTSGS 451
caNC      ISYLPSFFTKLEN-TSPMVKSKKQEIFRKLNSSGGGDSDMAAAQPGTEIFNLPAVTTSGS 452
muNC      ISYLPSFFTKLES-TSPMVKSKKQEIFRKLNSSGGGDSDMAAAQPGTEIFNLPAVTTSGA 449
ggNC      ISYLPSFFTKLEN-TSPMVKSKKQEIFRKLNSSGGGDSDMATAQPGTEIFNLPAVTTSGA 452
xtNC      ISYLPSFFTKLEN-TSPMVMYKKQEIFRKLNSSGGGDSEMAAAQPGTEIFNLPAVTTSGA 433
huSM      TSQPPAFFSKLTESNSAMVKSKKQEINKKLNTHNRNEPEYNISQPGTEIFNMPAITGTGL 464
caSM      TSQPPAFFSKLTESNSAMVKSKKQEINKKLNTHNRNEPEYNIGQPGTEIFNMPAITGAGL 464
muSM      TSQPPAFFSKLTESNSAMVKSKKQEINKKLNTHNRNEPEYNIGQPGTEIFNMPAITGAGL 460
ggSM      NTQPPAFFSKLTESNSAMVKSKKQEIIKKLSTTNKNETEYNVGQPGTEIFNMPAITGAGL 462
xlSM      NTQPPAFFSKLTESNSAMVKSKKQEMIKKLSTTNRSEQEYSVGQPGTEIFNMPANTGAGL 447
           :  *:**:** . .*.**  ****: :**.: . .: :   .********:** * :*

                          Motif 5
huNC      VSSRGHSFADPAS-NLGLEDIIRKALMGSFDDKVEDHGVVMSQPMGVVPGTANTSVVTSG 510
caNC      VSSRGHSFADPAS-NLGLEDIIRKALMGSFDDKVEEHGVVMSQPVGVVPGGANTSVVTSG 511
muNC      VSSRSHSFADPAS-NLGLEDIIRKALMGSFDDKVEDHGVVMSHPVGIMPGSASTSVVTSS 508
ggNC      VSSRGHSFADPAS-NLGLEDIIRKALMGNFDDKSEDHGVVMSQSIAVAPGNSSAVVSASN 511
xtNC      ISSRGHSFADPAS-NLGLEDIIRKALMGNFDDKSEDH----SVLVGVAQGNPSG--TQNS 486
huSM      MTYRSQAVQEHASTNMGLEAIIRKALMGKYD-QWEESPPLSANAFNPLNASASLP-AAMP 522
caSM      MTCRSQAVQEHASTNMGLEAIIRKALMGKYD-QWEE-PPLSANAFNPLNASASLP-AAIP 521
muSM      MTCRSQAVQEHASTNMGLEAIIRKALMGKYD-QWEEPPPLGANAFNPLNASASLPAAAMP 519
ggSM      ISCRSQSVQENSSTNMGLEAIIRKALMGKYDEQWEERSPLSANAFNSLNASASLP-AAMP 521
xlSM      ISSRNQTVPETSNSNMGLEAIIRKALMGKYDEHWDDRSALNANAFNPLNASSSLP-SAIP 506
          :: *.::. : :. *:*** ********.:* : ::     :  .    . ..
```

```
                               44   45                               Motif 6
huNC        ETRRE-EGDPSPHSGG-VCKPKLISKSNSRKSKSPIPG-QGYLGTERPSSVSSVHSEGDY 567
caNC        ETRRE-EGDPSPHSGG-VCKPKLISKSNSRKSKSPIPG-QGYLGTERPSSVSSVHSEGDY 568
muNC        EARRD-EGEPSPHAG--VCKPKLINKSNSRKSKSPIPG-QSYLGTERPSSVSSVHSEGDY 564
ggNC        ETRRE-EANPSPNSGGAVSKQKLIGKSNSRKSKSPIPG-QGYLGTERPSSVSSVHSEGDY 569
xtNC        EARRE-EANPSPNSGGTHKQKLISKYGSRKTKSPISGSQTYLGAERPSSVSSVHSEGDY 545
huSM        ITAADGRSDHTLTSPGGGGKAKVSGRPSSRKAKSPAPG---LASGDRPPSVSSVHSEGDC 579
caSM        ITAADGRSEHALTSPGGGGKAKVSGRPSSRKAKSPAPG---LASGDRPPSVSSVHSEGDC 578
muSM        ITTADGRSDHALTSPGGGGKAKVSGRPSSRKAKSPAPG---LASGDRPPSVSSVHSEGDC 576
ggSM        ITPADGRNEDVRSLPAGGGKPKIAARPNSRKAKSPAPG---LSSGERPPSVSSVHSEGDC 578
xlSM        ITTVDGRNDDMQSL-TGSGKAKIP-RPSSRKAKSPAPG---QASGERPPSASSVHSEGDC 561
             :  : . :          * *:  : .***:*** .*     . :**.*.********

                     Motif 7 45   46
huNC        HRQTPGW--AWEDRPSSTGSTQFPYNPLTMRMLSSTPPTPIACAPSAVNQAAPHQQNRIW 625
caNC        HRQTPGW--AWEDRPSSTGSTQFPYNPLTMRMLSSTPPTPIACAPSSVNQAAPHQQNRIW 626
muNC        HRQTPGW--AWEDRPSSTGSTQFPYNPLTIRMLSSTPPTQIACAPSAITQAAPHQQNRIW 622
ggNC        HRQTPVW--AWEDRPSSTGSTQFPYNPLTMRMLSSTPPTSIACAPPSMSQATTHQQNRIW 627
xtNC        -RQASAW--AWEDRPSSTGSTQFPYNPLTMGMLNSTPPSSMSCAPTSMTQTSAHQQSRIW 602
huSM        NRRTPLTNRVWEDRPSSAGSTPFPYNPLIMRLQAGVMASPPPPG-LPAGSGPLAGPHHAW 638
caSM        NRRTPLTNRVWEDRPSSAGSTPFPYNPLIMRLQAGVMASPPPPG-LPAGSGPLAGPHHAW 637
muSM        NRRTPLTNRVWEDRPSSAGSTPFPYNPLIMRLQAGVMASPPPPG-LAAGSGPLAGPHHAW 635
ggSM        NRRTPLTNRVWEDRPSSAGSTPFPYNPLTMRLPSGVVTAAPAVP-LPQGTP--GGQHHAW 635
xlSM        NRKTPLTNRVWEERPSSTGSTPFPYNPLTIRLPTGLVAAQTSSS-IPQSNS--GTPQRAR 618
             *::.    .**:****:*** ****** : :  .  .:  .    .          :

              Motif 8
huNC        EREPAPLLSAQYETLSDSDD 645
caNC        EREPAPLLSAQYETLSDSDD 646
muNC        EREPAPLLSAQYETLSDSDD 642
ggNC        EREPAPLLSAQYETLSDSDD 647
xtNC        EREPAPLLSEQYETLSDSDE 622
huSM        DEEPKPLLCSQYETLSDSE- 657
caSM        DEEPKPLLCSQYETLSDSE- 656
muSM        DEEPKPLLCSQYETLSDSE- 654
ggSM        DEEPKPLLCSQYETLSDSE- 654
xlSM        NEEPKPLLCSQYETLSDSE- 637
            :.** ***. ********:
```

Supplementary Figure 2
The progressive acquisition of motifs

	Motif 1 (L H L / IxxIIxxxI)	Motif 2 (L H L / IxxIIxxxI)
HsNC	ELRTRGKTTITAANFIDVIITRQI	RLITLADHICQIITQD
XtNC	ELRMRGKTTITAANFIDVIITQQI	RLITLADHICQIITQD
HuSM	ELRSLGKTTLTAATFIDAIIMRQI	RVVTLAQHISEVITQD
XlSM	ELRSLGKTTMTAANFIDAIIMRQI	RVVTLAQHISEVITQD
Cio	ELKVKGRATMTPEYLINAIIDQSI	VTKTLHEHSQDVISDI
Amph	PPPQGGQGPLTAANLIDAIITHQI	QQITLGEHISNIIHQD
Acor	EPGKKSKPSLTAANLIDVIITRSI	RPITLGEHIDSIISQD
Urch	EDPGQG-STFTAANLIDAIITRSI	RTVTLAEHIHSIVSLG

	Motif 3	Motif 4
HsNC	PSFFTKLEN-TSPMVKSKKQEIFRK	QPGTEIFNLPAVT
XtNC	PSFFTKLEN-TSPMVMYKKQEIFRK	QPGTEIFNLPAVT
HsSM	PAFFSKLTESNSAMVKSKKQEINKK	QPGTEIFNMPAIT
XlSM	PAFFSKLTESNSAMVKSKKQEMIKK	QPGTEIFNMPANT
Ciona	PGFMSKPVPTNEA-TKRKQKTPISG	QPGTRMGNVQLQH
Amph		QPGSEQWQPRPPQ
Acor		QPESGSSADQHKS
Urch		

	Motif 5 (L H L / IxxIIxxxI)	Motif 8
HsNC	GLEDIIRKALMG	EPAPLLSAQYETLSDSDD
XtNC	GLEDIIRKALMG	EPAPLLSEQYETLSDSDE
HsSM	GLEAIIRKALMG	EPKPLLCSQYETLSDSE-
XlSM	GLEAIIRKALMG	EPKPLLCSQYETLSDSE-
Ciona	SFELLIRKALIE	ERVPPLVTQYEPLSDSD*
Amphi	NLEDIIRKALMG	EPAPLLSSQYEPLSDTDEES*
Acor	SIEDVIDAT-MG	EPAPLLSSQYETLSDIESD*
Urch	PDTSLDRKA-MY	EPKPILSEQYETLSDSD*

Alignment of NCoR family motifs for human, Xenopus, sea-squirt, amphioxus and sea urchin. Blue = conserved residues, purple = NCoR1 indicative residues, red = NCoR2 indicative residues. Grey shading indicates restricted sequence similarity. Yellow box indicate identity to the consensus CoRNR box motif shown above. Red dots are above or below residues that do not conform to the CoRNR box consensus.

Supplementary figure 3

The unusual arrangement of Medaka NCoR2 exon 37

A. Alignment of Medaka, Xenopus and Zebrafish NCoR2 exon 37 nucleotide sequence around the internal exon splice donor that defines exon 37b in Xenopus. Whilst there is extensive homology in exon 37 and around the splice donor (red gt), there is little homology between the Medaka sequence and the Xenopus and Zebrafish sequences in exon 37b. The Medaka sequence contains an in-frame stop codon (orange) that would truncate the protein if the internal splice donor was not used. The 3' end of exon 37b in Xenopus and Zebrafish is flanked by the terminal exon splice donor (green gt).

```
            Exon 37                                Exon37b
Medaka     gcgattgctattcaggaacaggagctcagatcactcggtaagaccctataaccagctcag
            |  || | || ||||||||||||||| |||||||||||||||||       | ||
Xenopus    cccttttccatgcaggaacaggagctccgatcactcggtaagaccaccatgactgcggcc
           |   | ||||| |||||||| || || ||| |||||||||||||||||||||| ||||||
Zebrafish  gtgatgtccatacaggaacacgaacttcgagcactcggtaagaccaccatgacagcggcc


Medaka     atcaagagaagcacgtttttaagctctggagaaaaaaaaccaatcattcactattggtta
           | |   | |  | |  || | |    |  |            | |    |         |
Xenopus    aacttcatagacgccattatcatgcgtcaaatttcttgcgataacggaaagcgagaaaga
           ||||||||| |||| || ||||||| |||||||||||| |||   |  | || ||| |
Zebrafish  aacttcataaacgcgataatcatgcatcaaatttcttgtgatgcggcgatgccagagact


Medaka     gttgtctggttcatagcaactgggcttaaaagggtta
           |           || |   |      | |  |    |
Xenopus    ggctcgctaaatatcgacgccaatagtgatggtaaga
           ||  ||||    | |  || ||   ||||||||||||
Zebrafish  ggtgcgctcgcgaccaacggcacctgtgatggtaaga
```

B. Alignment of Medaka, Xenopus and Zebrafish SMRT protein sequences in the region encoded by exon 37 and 37b. The stop codon (red dash) in Medaka eliminates the CoRNR box encoded by exon 37b. Identity shown in grey shading, the boundary between residues encoded by exon 37 and 37b is marked by the red glycine (G).

```
Medaka      AIAIQEQELRSLGKTL-
Xenopus     PFSMQEQELRSLGKTTMTAANFIDAIIMRQISCDNGKRERGSLNIDANSD
Zebrafish   VMSIQEHELRALGKTTMTAANFINAIIMHQISCDAAMPETGALATNGTCD
                                  CoRNR box
```

Supplementary Table 2

PCR primers used for cloning and analysis

Xenopus NCoR2 alternative splicing (Fig. 3)
F 5’ TCCATGCAAGAACAGGAG 3’
R 5’ AATGGCACGGTGACTGTT 3’

Zebrafish NCoR2 alternative splicing (Fig. 3 and Fig. 4)
F 5’ GCAGCAGCGCGTCAAGTAGGG 3’
R 5’ GGCGAGGGTCACCACTCTCTG 3’

Lamprey NCoR2 alternative splicing (Fig. 3)
F 5’ GGCAAGGAGCGACCACAGAC 3’
R 5’ GCTTTGATAATCTTTCGTTATG 3’

Splicing plasmid pTBNde1 flanking exons (Fig. 2)
F (2-3 alpha) 5’ CAACTTCAAGCTCCTAAGCCACTGC 3’
R (B2) 5’ CCAGGGTCACCAGGAAGTTGGTTAA 3’

Amplifying Xenopus NCoR1 exon 37 genomic fragment (Fig. 2)
F 5’ GGTTATGTAGTTCCTGCATGCTG 3’
R 5’ CCATATTTGGATCTTCATACC 3’

Amplifying Xenopus NCoR2 exon 37 genomic fragment (Fig. 2)
F 5’ GAGGACTCAGAGCAGCATTAC 3’
R 5’ TGCATTCAGGTACTGAACACC 3’

Site directed mutagenesis of NCoR1: control site
F 5’ CTATTCAGAAGCAGGTAAAGAAAGAGCAGCG 3’
R 5’ CGCTGCTCTTTCTTTACCTGCTTCTGAATAG 3’

Site directed mutagenesis of NCoR1: test site
F 5’ CTACGAATGAGAGGTAAGACCACTATCACTG 3’
R 5’ CAGTGATAGTGGTCTTACCTCTCATTCGTAG 3’

Amplifying zebrafish genomic fragment containing exon 37 (Fig. 5)
F 5’ CATATGCACTCAAACTGCTGTCTGAAGC 3’
R 5’ CATATGGCCAACCACCACAAGCGTGCAC 3’
